# Extraction, Identification and Antioxidant Activity of 3-Deoxyanthocyanidins from *Sorghum bicolor* L. Moench Cultivated in China

**DOI:** 10.3390/antiox12020468

**Published:** 2023-02-12

**Authors:** Yanbei Wu, Yali Wang, Zhengyan Liu, Jing Wang

**Affiliations:** China-Canada Joint Lab of Food Nutrition and Health (Beijing), School of Food and Health, Beijing Technology and Business University (BTBU), 11 Fucheng Road, Beijing 100048, China

**Keywords:** sorghum, 3-deoxyanthocyanidins, solvent extraction, Apigeninidin, Luteolinidin, bioactivity

## Abstract

3-deoxyanthocyanidins (3-DAs) are typical flavonoids found in sorghum bran, and they have received much attention in recent years owing to their bioactivities. To further enhance the resource utilization of sorghums cultivated in China, three brewing sorghum cultivars (Liaoza-48, Liaonuo-11, and Liaonian-3) commonly used in China were selected as raw materials for the construction of an extraction technology system for 3-DAs and the clarification of their structures and bioactivities. Based on single-factor experiments and response surface analysis, the optimized system for the extraction of 3-DAs from sorghum grains was as follows: a hydrochloric acid-methanol solution (1:100, *v*/*v*) was the extraction solvent, the solid-liquid ratio was 1:20 (g/mL), the extraction time was 130 min, and the temperature was 40 °C. This extraction system was simple and feasible. High performance liquid chromatography analysis suggested that the main monomeric compounds of the extracted 3-DAs were Apigeninidin (AE) and Luteolinidin (LE). Among the three selected sorghum grains, Liaoza-48 had the highest amount of AE (329.64 μg/g) and LE (97.18 μg/g). Antioxidant experiments indicated that the 3-DAs extracted from Liaoza-48 showed higher free-radical scavenging activities for DPPH, ABTS^+^, and hydroxyl radicals than those extracted from Liaonuo-11 and Liaonian-3. These results provide basic data and technical support for the high-value and comprehensive utilization of sorghums in China.

## 1. Introduction

Sorghum (*Sorghum bicolor* L. Moench) is the fifth largest food crop in the world [1], with multiple applications including its use as grain and forage, and its function in brewing and sugar production; it has been known as “the essence of five grains and the longest of all grains” since ancient times [2]. One of the major producers of sorghum, China is also the world’s top importer of the product [3]. There is strong demand for sorghum in the domestic market, of which the brewing industry occupies a large proportion, and the demand for sorghum is growing rapidly. However, in recent years, the lack of research on deep-processing products from sorghum and on its comprehensive utilization has created the dilemma of increased production without any improvement in the yield of edible sorghum, and this has caused its acreage to shrink year by year [4], which is detrimental to the development of China’s food industry. Therefore, it is necessary to research the high-value utilization of sorghum and its by-products.

**Figure 1:**
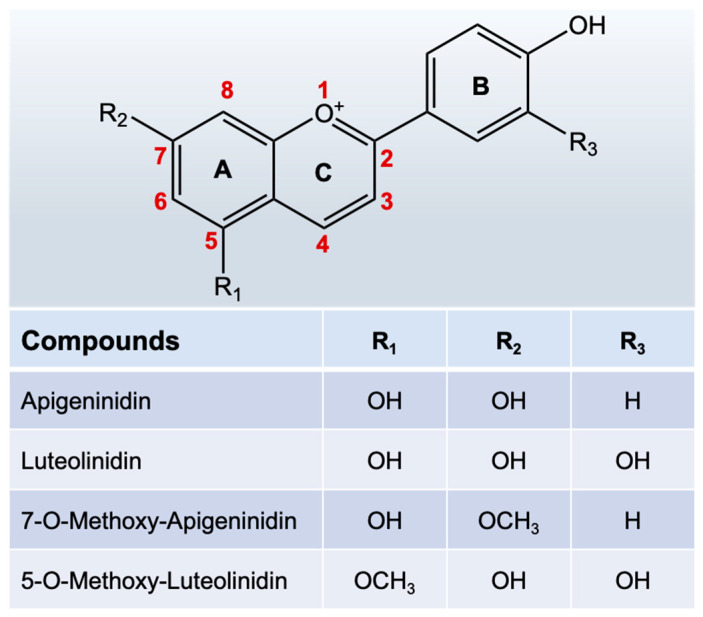
Structures of 3-DAs and their main derivatives in sorghum.

Sorghum grains are rich in many beneficial phytochemicals, e.g., 3-deoxyanthocyanidins (3-DAs), phenolic acids, and proanthocyanidins in addition to many nutrients [5]. Among these phytochemicals, 3-DAs are the most unique flavonoids in sorghum [6,7]. This class of compounds is extremely rare in other edible plants but is abundant in sorghum bran (up to 4.7–16 mg/g) [8]. Structurally, 3-DAs are analogs of anthocyanins, but they lack the hydroxyl group at the highly active C-3 position and exist mainly as free glycosyl ligands in sorghum, with better chemical stability and bioavailability than anthocyanins [9]. As previously reported, sorghum 3-DAs initially accumulate in plant cells as inclusion bodies and contain various monomers (e.g., Apigeninidin (AE), Luteolinidin (LE)) and their methoxy derivatives (Figure 1) as well as other metabolites, which are a class of small-molecule antimicrobial active substances produced by sorghum against external pathogenic infestation [10,11]. The content and composition of 3-DAs in sorghum are significantly affected by the genotype, pathogenic microorganisms, the environment, and genotype-environment interactions [12,13]. Taleon et al. evaluated the levels of 3-DAs in seven sorghum genotypes grown in four locations in Texas, USA, and four 3-DAs were detected and quantified using HPLC-DAD. The results showed that high levels of 3-DAs, especially methoxylated 3-DAs, were observed in red and lemon-yellow sorghums affected by weathering [14]. Dykes et al. investigated the effect of genotypes on the content of 3-DAs in 13 foreign sorghum varieties [15]. The results showed that the 3-DAs in sorghum with red/purple secondary plant color had the highest levels (32~680 μg/g), especially those with a black pericarp, which had higher levels of 3-DAs than those from tan plants. Additionally, some red sorghum genotypes turned black when exposed to sunlight, which caused a threefold increase in the levels of 3-DAs. Li et al. analyzed the soluble and insoluble phenolic compounds in 11 types of Chinese red sorghum, and found that four major 3-DAs in the soluble and insoluble fractions could be quantified, including LE, AE, 5-methoxy-luteolinidin, and 7-methoxy-apigeninidin [16]. Their total contents differed among red sorghum varieties and were found to be 8.36~76.45, 3.10~42.04, 3.03~22.72, and 1.61~7.27 μg/g, respectively. 

3-DAs have been found to exhibit excellent antioxidant, antibacterial, antiparasitic, and anti-inflammatory activities, thus showing the possibility of promising applications in the food, cosmetic, and pharmaceutical industries [17,18,19]. Importantly, Pearson’s correlation analysis suggested that 3-DAs might significantly contribute to the antioxidant capacities of sorghum, especially the soluble phenolic fractions [16]. Notably, sorghum 3-DAs from different sources may exhibit different bioactivities due to the differences in the content and substance composition of 3-DAs, thereby causing a biased understanding of their bioactivities. Thus, given the possible differences between Chinese and foreign sorghum cultivars caused by genotype and climatic conditions, the 3-DAs content, the compositions, and the bioactivities of different sorghums are thought to have distinctive features. At present, little research is focused simultaneously on the establishment of the extraction technology system and the clarification of the bioactivity of 3-DAs from sorghums cultivated in China, and this severely limits the scope of targeted guidance on the high-value utilization of Chinese sorghums. Thus, it is important to investigate the extraction, identification, and bioactivity of 3-DAs from representative high-yield Chinese sorghums in order to deepen the understanding of their high-value comprehensive utilization in China.

Based on the above-mentioned findings and hypothesis, in this work, three typical brewing sorghum grains (cultivars: Liaoza-48, Liaonuo-11, and Liaonian-3) commonly used in China were selected as raw materials for the construction of the extraction technology system for 3-DAs and the clarification of their compositions and bioactivities. Firstly, the effects of different extraction conditions (solvent system, solvent volume fraction, solid-liquid ratio, extraction time, and temperature) on the amount of 3-DAs extracted were investigated to optimize the solvent system for the extraction of 3-DAs. Furthermore, the extracts of different sorghum grains were identified by means of high-performance liquid chromatography (HPLC) to clarify their compositions. Finally, the antioxidant activities of the extracted 3-DAs were determined by evaluating their free-radical scavenging activities. This work can provide data support and theoretical references to help establish the extraction and utilization system for sorghum 3-DAs; this is beneficial for the development and comprehensive utilization of sorghum cultivated in China.

## 2. Materials and Methods

### 2.1. Materials

Sorghum grains (cultivars: Liaozha-48, Liaonuo-11, Liaonian-3) were provided by the Research Institute of Sorghum, Liaoning Academy of Agricultural Sciences (Liaoning, China). Methanol, anhydrous ethanol, and hydrochloric acid were of analytic grade and were obtained from Sinopharm Chemical Reagent Co., Ltd. (Shanghai, China). Acetone and acetonitrile were of chromatographic grade and were provided by MREDA Technology, Inc. (Beijing, China). Standard Apigeninidin and Luteolinidin were purchased from Shanghai Yuanye Bio-technology Co., Ltd. (Shanghai, China). Potassium peroxodisulfate was of analytical grade and was obtained from Damao Chemical Reagent Factory (Tianjin, China). Shanghai Aladdin Biochemical Technology Co., Ltd. (Shanghai, China) provided 1,1-diphenyl-2-trinitrophenylhydrazine (DPPH) and 2,2-azino-bis(ethylbenzene-thiazoline-6-sulfonic acid) (ABTS). 

### 2.2. Optimization of the Extract of 3-DAs from Sorghum Grains

#### 2.2.1. Pretreatment of Sorghum Grains

The selected sorghum grains were pretreated according to the procedure reported by Al-Rabadi et al. with a slight modification [20]. Sorghum grains first passed through a 10-mesh sieve to remove impurities and were then dried using an electric blast-drying oven (WG9220A, Tianjin Tongli Xinda Instrument Factory, Tianjin, China) at 60 °C for 4 h. Thereafter, the sorghum was ground through a high-speed multifunctional crusher. After passing through an 80-mesh sieve, the powder which emerged through the sieve was collected and stored in a desiccator for further use.

#### 2.2.2. Extraction of 3-DAs from Sorghum Grains

Single-factor experiments were first conducted to optimize the extraction process. Firstly, 0.5 g of sorghum grain powder was accurately weighed and placed in a centrifuge tube with a volume of 50 mL. Thereafter, the effects of different solvent volume fractions, solid-liquid ratios (the ratios of sorghum powder mass to solvent volume), extraction temperatures, and times on the amount of 3-DAs extracted from different sorghum grain powders were investigated by employing the 1% of hydrochloric acid-methanol extraction system and the acetone extraction system under certain conditions. The effects of each of these factors on the extraction effect were investigated individually and are shown in Table S1. The amount of 3-DAs extracted from different sorghum grain powders was used as the response value. 

Based on the results of single-factor experiments, three main factors affecting the amount of 3-DAs extracted were selected as independent variables, and the amount of 3-DAs extracted was used as the response value. A response surface Box-Behnken experimental design method was used to select the relatively best level and its two adjacent levels as the factors and levels of the orthogonal test (as shown in Table 1), and a three-factor, three-level L9 (3^3^) orthogonal test was conducted to optimize the parameters of the process conditions for solvent extraction of sorghum 3-DAs.

#### 2.2.3. Determination of 3-DAs in the Crude Extracts

The content of 3-DAs in sorghum was preliminarily determined using the pH difference method reported by Choi et al. [21] and Fuleki and Francis [22] with a slight modification. The crude extract of sorghum 3-DAs was diluted with a sodium acetate–acetate acid buffer solution (pH 1.0), and then it was scanned in the range from 200 to 800 nm using a UV-Vis spectrometer (WFJ72-721, Shanghai Spectrum Instruments Co., Ltd., Shanghai, China) to confirm the λvis-max (within the linear range of 0.2~1.2 on the spectrophotometer) and dilution factors of 3-DAs. Subsequently, distilled water was used as a blank control, and sodium acetate–acetate acid buffer solutions with pH of 1.0 and 4.5 were used to dilute the samples according to the previously determined dilution. Next, the absorbance values of diluted samples at λ_vis-max_ (483 nm) and 700 nm were recorded. The content of 3-DAs was calculated in terms of AE monomer in combination with Fuleki’s Equation (1):

(1) $$AE/\left(mg/g\right)=\frac{A\times Mw\times DF\times V}{\varepsilon \times m\times L}$$

where *A* = (*A*_483nm_ − *A*_700nm_)_pH1.0_ − (*A*_483nm_ − *A*_700nm_)_pH4.5_; *AE* is Apigeninidin; *Mw* is the relative molecular mass of *AE*, 255.24 g/mol; *DF* is the dilution factor and its value is the ratio between the volume after dilution and the volume before dilution; *ε* is the molar extinction coefficient: *AE* is 30,400 L × mol^−1^ × cm^−1^; *L* is the light range, cm; *V* is the total volume of extraction solution, mL; *m* is the mass of sorghum grain powder, *g*.

### 2.3. Identification of the Extracts

#### 2.3.1. Establishment of Standard Curves

1.0 mg of AE and LE were respectively weighed and dissolved in hydrochloric acid-methanol solution (1:100, *v*/*v*) to prepare a single standard stock solution with a concentration of 1 mg/mL after being shaken thoroughly, and then they were stored at −18 °C for further use. The above two single standard stock solutions were diluted step by step to prepare single standard working solutions with concentrations of 2.5 μg/mL, 25 μg/mL, 50 μg/mL, 100 μg/mL, 200 μg/mL, and 400 μg/mL and then subjected to HPLC (LC-20AT equipped with Agilent-ZORBAXSB-C18, 5 μm, 4.6 mm × 250.0 mm, Agilent, CA, USA) testing according to the method described by Yang et al. [23] with a slight modification. The test conditions were as follows. The column temperature was 35 °C, and the mobile phases were 0.1% of formic acid aqueous solution (A) and 0.1% of formic acid-acetonitrile solution (B). The flow rate was 1.0 mL/min, the detection wavelength was 480 nm, and the injection volume was 10.0 μL. The gradient elution conditions were as shown in Table S2. The standard curves were plotted with the peak areas of AE and LE. The vertical coordinates indicated the peak areas (*y*), and the horizontal coordinates indicated the concentrations of the standard solutions (*x*). 

The AE and LE blank samples (without extracted sorghum 3-DAs) were accurately configured at concentrations of 0.1 mg/mL, 0.2 mg/mL, and 0.5 mg/mL, respectively. The peak areas were determined by HPLC, and the recoveries and relative standard deviations (relative standard deviation, RSD) were calculated, and where the RSDs of both standards were less than 2.0%, the method was deemed to be of good accuracy.

#### 2.3.2. Identification of the Compositions of Extracted 3-DAs

The 3-DAs extracted from different sorghum grain species were tested by HPLC according to the above-mentioned conditions. Then, the monomeric compounds in the extracts of the 3-DAs from the three selected sorghum grains were identified in combination with the peak positions of the sample chromatograms and the peak positions of the standard chromatograms. Next, the content of AE and LE of the different 3-DAs was calculated by using the established standard curves. The contents of AE and LE in the extracted 3-DAs were calculated according to Equation (2):

(2) $$AE\;or\;LE\;content/\left(mg/g\right)=\frac{C\times V}{m}$$

where *C* is the concentration of *AE* or *LE* calculated from the standard curve, μg/mL; *V* is the total volume of extraction solution, mL; and *m* is the mass of sorghum grain powder, *g*.

### 2.4. Antioxidant Activity Assays

The DPPH radical scavenging activity was determined using the method reported by Motikar et al. [24] with a slight modification. 4.0 mg of DPPH was weighed and dissolved in anhydrous ethanol, then the solution volume was fixed to 50 mL with even mixing to prepare a DPPH solution with a concentration of 2 × 10^−4^ mol/L, and it was subsequently stored at −20 °C away from light. The three extract sample solutions were diluted to a certain multiple, and each diluted extract sample solution, DPPH solution, and anhydrous ethanol were accurately aspirated into three centrifuge tubes to obtain test solutions (labeled as S_0_, S_1_, and S_2_) according to the specific formula presented in Table S3. After standing for 30 min away from light, the absorbance values (*A*_0_, *A*_1_, and *A*_2_) of S_0_, S_1_, and S_2_ were parallelly measured three times at 517 nm, respectively. The DPPH radical scavenging activity was calculated according to Equation (3):

(3) $$DPPH\;scavenging\;activity/\%=\left(1-\frac{A_{2}-A_{1}}{A_{0}}\right)\times 100\%$$

where *A*_0_ is the absorbance of S_0_ containing DPPH solution and anhydrous ethanol; *A*_1_ is the absorbance of S_1_ containing anhydrous ethanol and extract sample solution; and *A*_2_ is the absorbance of S_2_ containing DPPH solution and diluted extract sample solution.

The ABTS^+^ radical scavenging activity was assessed using the method reported by Alejandra et al. [25] with a slight modification. Firstly, 0.0384 g of ABTS and 0.0134 g of potassium peroxodisulfate were accurately weighed and dissolved in distilled water. The volume of the two solutions was then fixed to 10 mL, respectively, to prepare 3.84 mg/mL of ABTS^+^ solution and 1.34 mg/mL of potassium peroxodisulfate solution. Afterwards, both of the solutions were mixed with a 1:1 volume ratio and left to stand for 16 h to obtain an ABTS^+^ stock solution. Before testing, the ABTS^+^ stock solution was diluted 20–30 times with a PBS buffer solution (pH = 7.4), and the diluted ABTS^+^ solution was measured at 734 nm to record the absorbance value (*A*_734 nm_ = 0.7 ± 0.02), which represented the absorbance value of ABTS^+^ solution. Thereafter, the three extract sample solutions were diluted by a certain multiple, and then they were employed to prepare the test solutions according to the specific formula shown in Table S4. After even mixing, the solutions were left to stand for 6 min to obtain test solutions (labeled as S_0_ and S_1_), and the absorbance values (*A*_0_ and *A*_1_) of S_0_ and S_1_ were parallelly measured three times at 734 nm. The ABTS^+^ radical scavenging rate was then calculated according to Equation (4):

(4) $${\mathrm{ABTS}}^{+}\;scavenging\;activity/\%=\frac{A_{0}-A_{1}}{A_{0}}\times 100\%$$

where *A*_0_ is the absorbance of S_0_ containing ABTS^+^ solution and ultrapure water and *A*_1_ is the absorbance of S_1_ containing ABTS^+^ solution and diluted extract sample solution.

The hydroxyl radical scavenging activity was directly determined using the assay kit (GRS-G0153W) provided by Suzhou Grace Biotechnology Co., Ltd. (Suzhou, China) with the sample addition volume of 150 μL.

### 2.5. Statistical Analyses

All the experiments were carried out three times, and the means and standard deviations were the average of repetitions. Statistical analysis was performed with the software Origin 8.0 (OriginLab Corporation, MA, USA). Results were analyzed by means of one-way analysis of variance (ANOVA) and differences were considered significant at *p* < 0.05.

## 3. Results and Discussion

### 3.1. Optimization of the Extraction of 3-DAs from Sorghum

#### 3.1.1. Single-Factor Analysis

**Figure 2:**
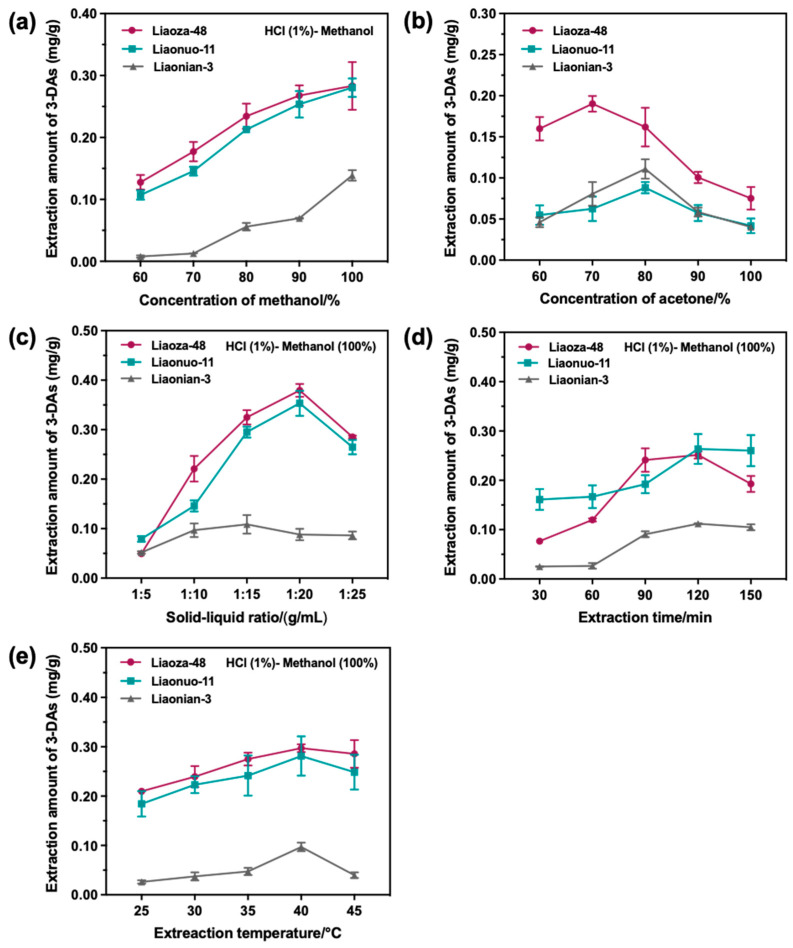
The effects of extraction conditions ((**a**): concentration of methanol; (**b**): concentration of acetone; (**c**): solid-liquid ratio; (**d**): extraction time; (**e**): extraction temperature) on the extracted amount of 3-DAs from different sorghum grains.

Firstly, the sorghum 3-DAs were extracted using different solvent systems at a solid-liquid ratio of 1:20 (g/mL), an extraction temperature of 40 °C, and an extraction time of 120 min. The solvent systems were hydrochloric acid (1%, *v*/*v*)-methanol (60%, 70%, 80%, 90%, 100%, *v*/*v*), and pure acetone (60%, 70%, 80%, 90%, 100%, *v*/*v*). As can be seen from Figure 2a, the effect of different volume fractions of methanol on the extraction of sorghum 3-DAs was more pronounced. The amount of 3-DAs extracted from all three sorghum grain species increased gradually with an increasing volume fraction of methanol and reached a maximum value using 1% of hydrochloric acid and 100% of methanol. For the acetone solvent system (Figure 2b), the extraction amounts of 3-DAs from Liaonuo-11 and Liaonian-3 showed a trend of first increasing and then decreasing. When the volume fraction of acetone exceeded 80%, the amount of 3-DAs extracted decreased sharply with the further increase of acetone concentration, while the amount of 3-DAs extracted in Liaoza-48 reached the maximum at 70% of acetone. This may be due to the high-volume fraction of acetone, which reacted with anthocyanin to form pyranocyanin and reduced the amount of detectable 3-DAs [26,27]. Considering the actual extraction yield, 1% of hydrochloric acid-100% of methanol was selected as the optimal extraction solvent system for the extraction of sorghum 3-DAs.

Subsequently, the effect of the solid-liquid ratio (1:5, 1:10, 1:15, 1:20, and 1:25 g/mL) on the extraction of 3-DAs was investigated under the following conditions. The solvent system was 1% of hydrochloric acid-100% of methanol, the extraction temperature was 40 °C, and the extraction time was 120 min. As illustrated in Figure 2c, the amount of 3-DAs extracted from Liaoza-48 and Liaonuo-11 was significantly affected by the solid-liquid ratio, which significantly increased when raised from 1:5 to 1:20 and then decreased with the increase in the solid-liquid ratio. For Liaonian-3, the amount of 3-DAs extracted was not obviously affected by the solid-liquid ratio, and the highest amount of 3-DAs extracted was achieved at the solid-liquid ratio of 1:15. The above results suggested that an excessively low solvent dosage would cause a low extraction efficiency of 3-DAs, resulting in a low extraction amount, while an excessively high solvent dosage would cause side reactions leading to the loss of 3-DAs. Therefore, when other conditions were fixed, the solid-liquid ratio of 1:20 (g/mL) was preferably selected for the extraction of sorghum 3-DAs.

Subsequently, the effect of extraction time (30, 60, 90, 120, and 150 min) on the extraction of 3-DAs was investigated under the following conditions. The solvent system was 1% of hydrochloric acid-100% of methanol, the solid-liquid ratio was 1:20 (g/mL), and the extraction temperature was 40 °C. As shown in Figure 2d, the extraction time had a significant effect on the amount of 3-DAs extracted from Liaoza-48, while it had a moderate impact on the amount of 3-DAs extracted from Liaonuo-11 and Liaonian-3. When the extraction time exceeded 120 min, the amount of 3-DAs extracted from the three sorghum grain species slightly decreased owing to the increased degree of oxidation of the 3-DAs and the simultaneous leaching of macromolecular solids [28,29]. Therefore, when other conditions were fixed, the extraction time of 120 min was preferably selected to extract sorghum 3-DAs.

Finally, the effect of extraction temperature (25, 30, 35, 40 and 45 °C) on the amount of 3-DAs extracted was investigated under the following conditions. The extraction solvent system was 1% of hydrochloric acid-100% of methanol, the solid-liquid ratio was 1:20 (g/mL), and the extraction time was 120 min; the results are shown in Figure 2e. It was found that the amount of 3-DAs extracted increased slowly as the extraction temperature was raised, and it reached the highest value when the extraction temperature was 40 °C for all sorghum grains. When the extraction temperature exceeded 40 °C, the amount of 3-DAs extracted decreased, which may be caused by the instability or decomposition of anthocyanins at excessively high extraction temperatures [30,31]. Therefore, when other conditions were fixed, the extraction temperature was preferably set to 40 °C for the extraction of sorghum 3-DAs.

#### 3.1.2. Response Surface Analysis

Based on the results of single-factor experiments, three main factors affecting the amount of 3-DAs extracted, including methanol volume fraction (A), solid-liquid ratio (B), and extraction time (C), were selected as independent variables. The optimal extraction solvent system chosen; this was 1% of hydrochloric acid-methanol. The amount of 3-DAs extracted was used as the response value, and Liaoza-48 was selected as the representative variety owing to its higher 3-DAs content. Based on the experimental results of the effects of single factors on the amount of 3-DAs extracted, a Box-Behnken central combination test was conducted using the factor and level design in the orthogonal test design table (Table 1). There were 17 test sites, including 5 central pilots and 12 analytic pilots (Table 2). These results were further analyzed using Design-Expert 12.0.3 software to obtain quadratic polynomial regression equations (as shown below) for the extraction of sorghum 3-DAs.

Y = 0.2760 + 0.0419*A* − 0.0206*B* + 0.0128*C* + 0.0136*AB* + 0.0098*AC* − 0.0119*BC* − 0.0109*A*^2^ − 0.0422*B*^2^ − 0.0456*C*^2^

ANOVA was performed on the above regression model, and the results are presented in Table 3; these results indicated that the linear correlation of the regression model was highly significant (*p* < 0.0001). The R^2^ (0.9787) of the regression model and the lack of fit *p*-value (0.0539) was higher than 0.05, indicating that it was not significant, and the regression model adequately fitted the experimental results [32]. Thus, this regression model could be used to analyze and predict the process results of the extraction of sorghum 3-DAs by means of the established solvent system. In the regression model, the primary term factors A and B were highly significant (*p* < 0.01), the factor C and AB interaction terms were significant (*p* < 0.05), and the secondary terms B and C were both highly significant (*p* < 0.01). By comparing the *F*-values, it can be seen that the degree of influence of the three single factors on the amount of 3-DAs extracted was in the following order: methanol volume fraction > solid-liquid ratio > extraction time.

**Figure 3:**
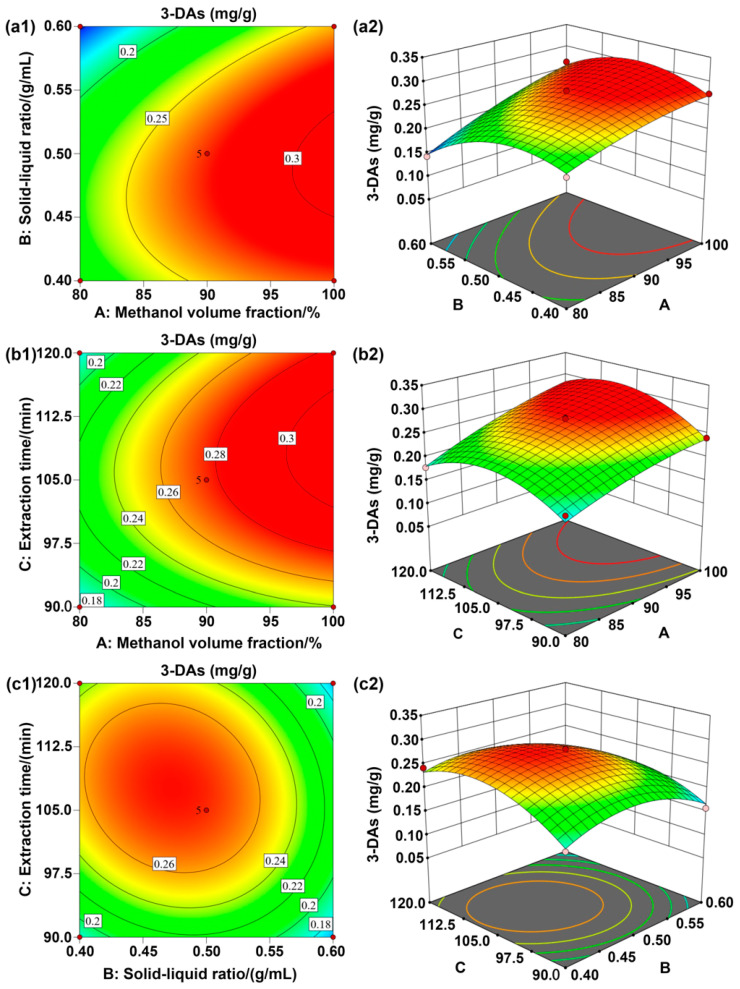
Response surface graphs of the effects of the three influencing factors (A: methanol volume fraction, %; B: solid-liquid ratio, g/mL; C: extraction time, min) on the amount of 3-DAs extracted from sorghum ((**a**): the interaction between A and B; (**b**): the interaction between A and C; (**c**): the interaction between B and C).

The interaction between independent variables was investigated using contour plots that were drawn between two variables (methanol volume fraction and solid-liquid ratio, solid-liquid ratio and extraction time, and methanol volume fraction and extraction time), while the other variables were kept constant. All the variables used in this work showed both positive and negative effects in their quadratic terms. Figure 3a illustrates that the interaction between the methanol volume fraction and the solid-liquid ratio was significant, while the interaction between the solid-liquid ratio and extraction time was not significant. It can also be seen from Figure 3 that the factor showing the highest effect on the amount of 3-DAs extracted from sorghum grain was the methanol volume fraction, followed by the solid-liquid ratio; the extraction time showed the lowest effect on the extraction process. This was consistent with the result of the regression analysis presented in Table 3. According to the prediction of Design Expert software, the amount of 3-DAs extracted reached the maximum by a combination of coded levels at the following conditions: extraction solvent system was 1% of hydrochloric acid-99.99% of methanol, the solid-liquid ratio was 1:19.3 (g/mL), the extraction time was 129.6 min, and the extraction temperature was 40 °C. In such conditions, the predicted response of the amount of 3-DAs extracted would be 0.31 mg/g. To validate the above prediction, the experiment was repeated three times under slightly modified conditions: the solvent was 1% of hydrochloric acid-100% of methanol, the solid-liquid ratio was 1:20 (g/mL), the extraction time was 130 min, and the extraction temperature was 40 °C. The amount of sorghum 3-DAs extracted could be 0.33 mg/g, which was close to the predicted value (0.31 mg/g). This result validated the accuracy of the experimental design in this work, showing that the optimized process was reliable. Given the similarity in the effects of extraction conditions on the amount 3-DAs extracted from the three selected sorghum grain species, this optimized process further served as the representative extraction process for Liaonuo-11 and Liaonian-3.

### 3.2. Identification of the Extracts

#### 3.2.1. Identification of the Main Compounds in the Extracts

**Figure 4:**
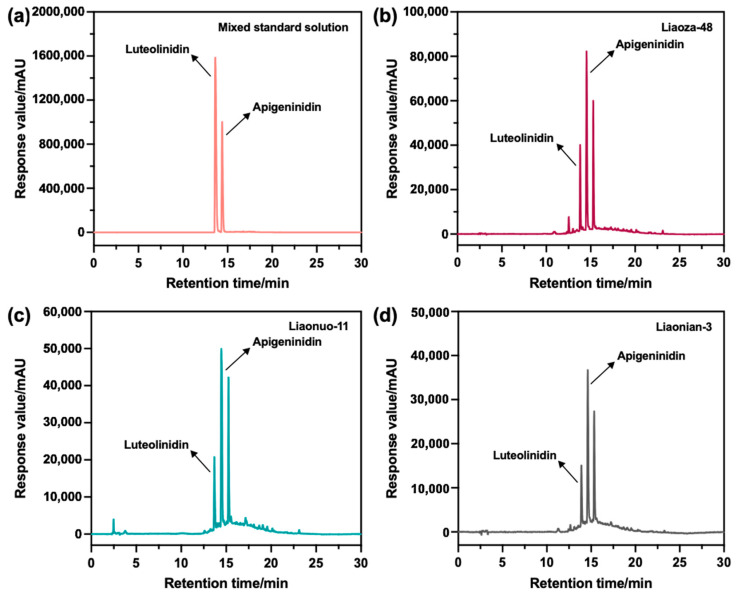
HPLC chromatograms of the standard Apigeninidin and Luteolinidin (**a**), as well as the extracts from different sorghum grain species ((**b**): Liaoza-48; (**c**): Liaonuo-11; (**d**): Liaonian-3).

As previously reported, Apigeninidin (AE), Luteolinidin (LE), and their methoxy derivatives are the main monomeric forms of sorghum 3-DAs, which are responsible for the bioactivity of 3-DAs [33]. To facilitate a fuller understanding of the monomeric components of 3-DAs, the crude extracts of 3-DAs from the three sorghum grains were further separated, purified, and characterized by HPLC. Under the chromatographic conditions described above, the monomeric components of the 3-DAs were well separated (Figure 4). Compared with the chromatographic curve of standard AE and LE, the chromatographic curves of the 3-DAs extracted from Liaoza-48, Liaonuo-11, and Liaonian-3 showed the typical peaks of AE and LE, indicating these extracted 3-DAs contained monomeric AE and LE. Notably, in addition to the above-mentioned typical peaks, there was another distinct peak for the three extracted 3-DAs, which might be ascribed to 7-Methoxy-Apigeninidin, according to the work reported by Yang et al. [23]. In view of the accuracy of the identification of the extracts, AE and LE were used as the typical substances for clarifying the specific monomer content in the extracted 3-DAs.

#### 3.2.2. The Content of the Main Compounds in the Extracts

**Figure 5:**
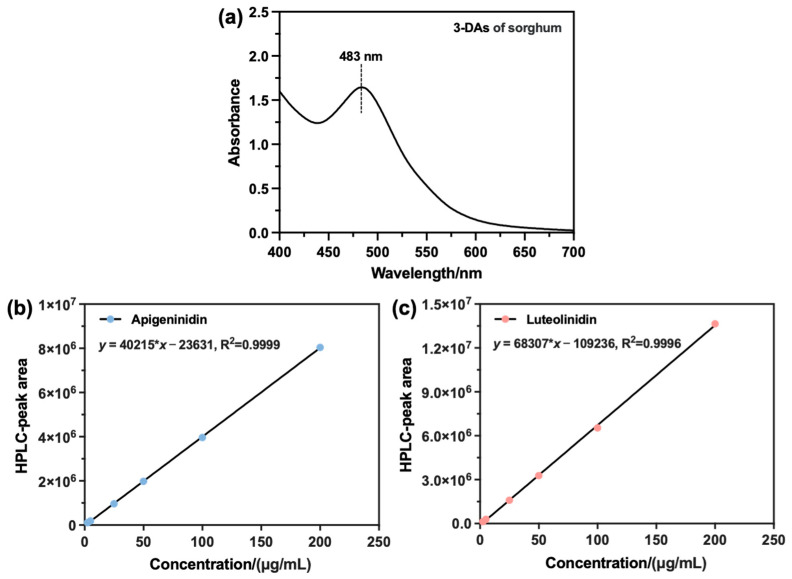
UV-Vis spectrum of the extract (**a**) and the standard curves of Apigeninidin (AE) (**b**) and Luteolinidin (LE) (**c**).

**Figure 6:**
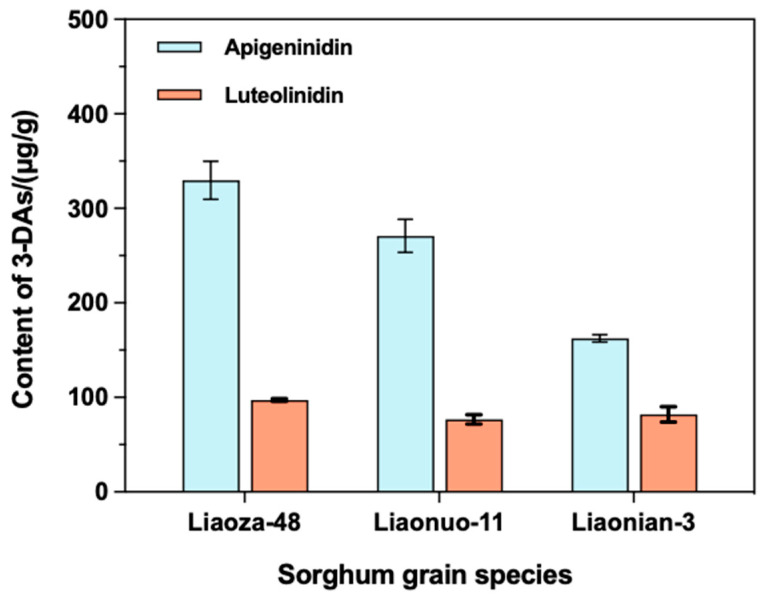
The content of 3-DAs of the three sorghum grain species in the forms of AE and LE.

Firstly, the UV-Vis spectrum of the extracted sorghum 3-DAs was recorded, and the spectrum in the 400–700 nm region is shown in Figure 5a. The extracted sorghum 3-DAs had a maximum absorption peak at 483 nm in the visible region. Given this, the HPLC measurements of standard AE, LE, and the extracted 3-DAs were subsequently performed at this wavelength. Figure 5b,c show the standard curves of AE and LE, respectively. It can be seen that the linearity of the standard curves of both AE and LE was good. Moreover, the recovery rate of the components of the 3-DAs in sorghum presented in Table S4 also showed that the recoveries of the two components were in the range of 96%~100%. The RSDs were 1.5% for AE and 1.3% for LE (Table S5), both of which were less than 2.0%, indicating a good testing accuracy of the established method. Based on this, the specific contents of AE and LE in the extracted 3-DAs were further determined and shown in Figure 6. It can be observed that the 3-DAs extracted from Liaoza-48 had the highest content of AE (329.64 μg/g) and LE (97.18 μg/g). The 3-DAs extracted from Liaonuo-11 had an AE content of 270.93 μg/g and an LE content of 76.84 μg/g. For the 3-DAs extracted from Liaonian-3, the content of AE and LE was only 162.50 μg/g and 82.05 μg/g, respectively. In light of these results, it can be concluded that Liaoza-48 is a more suitable raw material for the extraction of bioactive 3-DAs than the other two sorghum grains.

### 3.3. Antioxidant Activity of the Extracts

**Figure 7:**
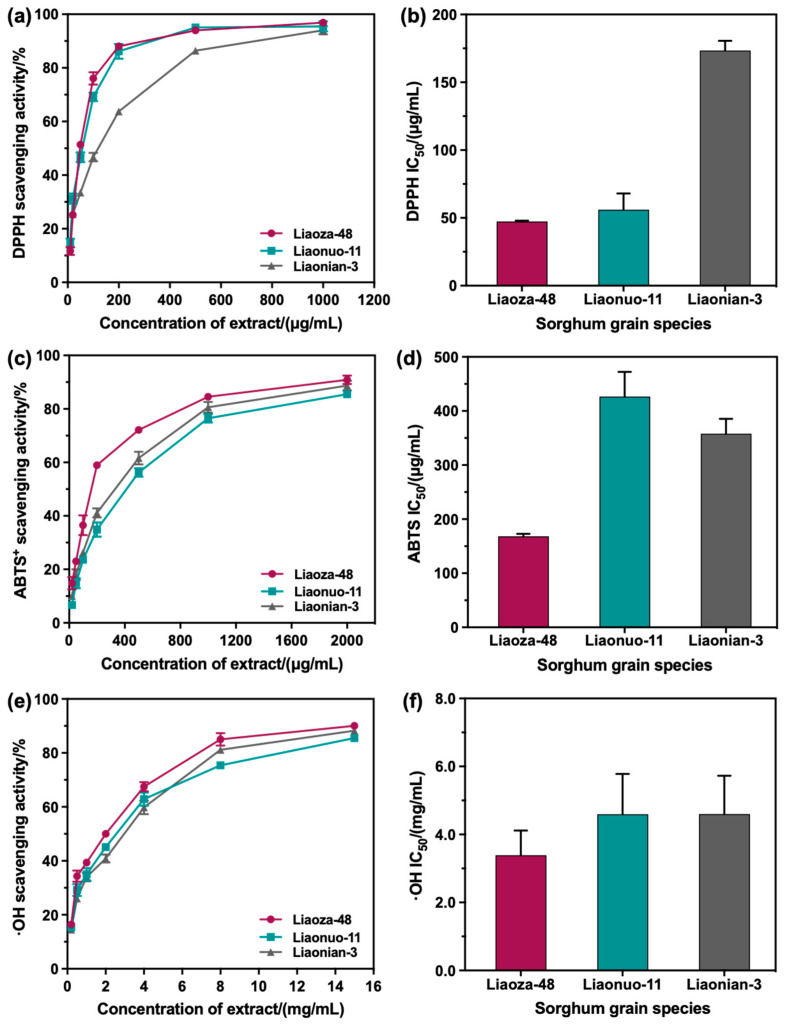
The free-radical scavenging activity of 3-DAs in three sorghums ((**a**): DPPH; (**c**): ABTS^+^; (**e**): •OH) and the IC_50_ values of different free-radical scavenging activities ((**b**): DPPH; (**d**): ABTS^+^; (**f**): •OH).

Free-radical scavengers play important roles both in food products and in the human body by counteracting oxidation processes [34]. Recently, an increasing number of researches have focused on exploring the free-radical scavengers for foods [35]. As reported by Carbonneau, the sorghum extracts containing 3-DAs were effective at preventing low-density lipoprotein vitamin E depletion and conjugated diene production, exhibiting antioxidant capacity [7]. Therefore, the free-radical scavenging activities (FRSAs) of DPPH, ABTS^+^, and the hydroxyl radicals of the 3-DAs extracted from Liaoza-48, Liaonuo-11, and Liaonian-3 were further determined to evaluate the antioxidant activity of extracted sorghum 3-DAs as a means of clarifying their potential as free-radical scavengers. Firstly, appropriate amounts of sorghum 3-DAs extracted from Liaoza-48, Liaonuo-11, and Liaonian-3 were diluted to 10 μg/mL, 20 μg/mL, 50 μg/mL, 100 μg/mL, 200 μg/mL, 500 μg/mL, and 1000 μg/mL, respectively. The scavenging activity of DPPH radicals at different concentrations of the extracted 3-DAs was determined. As shown in Figure 7a, it can be seen that the DPPH radical scavenging activity of the 3-DAs extracted from the three sorghum grain species increased as the concentration of the extracted 3-DAs increased. Among them, the DPPH radical scavenging activity was similar in the 3-DAs extracted from Liaoza-48 and Liaonuo-11 and higher than that of the 3-DAs extracted from Liaonian-3. Figure 7b illustrates that the IC_50_ values of the three sorghum 3-DAs extracted were 47.42 μg/mL for Liaoza-48, 56.13 μg/mL for Liaonuo-11, and 173.43 μg/mL for Liaonian-3, respectively. As we know, a lower IC_50_ value indicates better free-radical scavenging ability [36]. Thus, it can be concluded that the DPPH scavenging ability of the 3-DAs extracted from Liaoza-48 was the highest, while that of the 3-DAs extracted from Liaonian-3 was the lowest. 

Next, appropriate amounts of sorghum 3-DAs extracted from Liaoza-48, Liaonuo-11, and Liaonian-3 were diluted to 20 μg/mL, 50 μg/mL, 100 μg/mL, 200 μg/mL, 500 μg/mL, 1000 μg/mL, and 2000 μg/mL, respectively, and the scavenging activities of ABTS^+^ radicals at different concentrations were determined. As illustrated in Figure 7c, the ABTS^+^ radical scavenging activity of the 3-DAs extracted from the three sorghum grain species increased as the concentration of the extracted 3-DAs increased. The ABTS^+^ radical scavenging activity of the 3-DAs extracted from Liaoza-48 was higher than that of the 3-DAs extracted from Liaonuo-11 and Liaonian-3. The IC_50_ values of the three extracted sorghum 3-DAs were 168.3.79 μg/mL for Liaoza-48, 426.6 μg/mL for Liaonuo-11, and 357.87 μg/mL for Liaonian-3, respectively (Figure 7d). Thus, it can be concluded that the ABTS^+^ scavenging ability of the 3-DAs extracted from Liaoza-48 was the highest, being considerably higher than that of the 3-DAs extracted from Liaonuo-11 and Liaonian-3, while that of the 3-DAs extracted from Liaonuo-11 was the lowest. 

As we know, the hydroxyl radical is a most reactive radical that can attack and damage almost every macromolecule in a living cell [37]. The hydroxyl radical scavenging activity of the 3-DAs extracted from Liaoza-48, Liaonuo-11, and Liaonian-3 was determined using extract concentrations of 0.2 mg/mL, 0.5 mg/mL, 1 mg/mL, 2 mg/mL, 4 mg/mL, 8 mg/mL, and 15 mg/mL, respectively. Figure 7e illustrates that the hydroxyl radical scavenging activity of the 3-DAs extracted from the three sorghum grain species shows a variation trend similar to the ABTS^+^ radical scavenging activity. Figure 7f shows that the IC_50_ values of 3-DAs extracted from Liaonuo-11 (4.60 mg/mL) and Liaonian-3 (4.60 mg/mL) were similar and were higher than the IC_50_ value of the 3-DAs extracted from Liaoza-48 (3.39 mg/mL), suggesting that the 3-DAs extracted from Liaoza-48 had the highest hydroxyl radical scavenging activity among the extracts of the three selected sorghum grain species. 

When compared with the antioxidant properties of the six sorghum extracts reported by Kumari et al. [38], the DPPH and ABTS^+^ results of the extracts of Liaoza-48 and Liaonuo-11 in the present work were found to be higher than all of them and were also much higher than those in the previous data shown by Zhu et al. with an IC_50_ value of 115.77 ± 9.01 μg/mL in the sorghum variety Jinza No.15 [39]. The DPPH results of the selected three Chinese sorghum grain species were all higher than the highest DPPH activity of decorticated sorghum grains DSOR 33 with an IC_50_ value of 236.0 ± 1.98 μg/mL reported by Ofosu et al. [40]. However, for the ABTS^+^ result, only the antioxidant property of the extract of Liaoza-48 was higher than the highest ABTS^+^ radical scavenging activity of decorticated sorghum grains DSOR 11 with an IC_50_ value of 317.05 ± 1.06 μg/mL [40]. Shen et al. investigated the hydroxyl radical scavenging activity of an extract of black highland barley (BHLPE) [37]. It was found that the IC_50_ value of BHLPE was 415.27 μg/mL, indicating that BHLPE was an effective scavenger of hydroxyl radicals. In comparison, the hydroxyl radical scavenging activities of the extracts of the selected three Chinese sorghum grain species were undesirable. In summary, the 3-DAs extracted from the three sorghum grain species had favorable antioxidant capabilities to scavenge the DPPH, ABTS^+^, and hydroxyl radicals. The 3-DAs extracted performed the best scavenging activity for the DPPH radical. Moreover, the 3-DAs extracted from Liaoza-48 showed the most favorable scavenging activities for all the selected free radicals. These results might be closely related to the content of 3-DAs in the extracts of sorghum grain species.

## 4. Conclusions

In this work, the extraction efficiency of 3-deoxyanthocyanidins (3-DAs) from the grain of three typical sorghum cultivars (Liaoza-48, Liaonuo-11, and Liaonian-3) in China was investigated under two different solvent systems (1% of hydrochloric acid-methanol and acetone). The optimized extraction system was constructed, and the specific extraction conditions were as follows. The extraction solvent was composed using 1% of hydrochloric acid and 100% of methanol. The solid-liquid ratio was controlled at 1:20 (g/mL), the extraction time was 130 min, and the temperature was 40 °C. Under such conditions, the amount of 3-DAs extracted could be 0.33 mg/g for the representative Liaoza-48 grain. In addition, the compositions of the 3-DAs extracted from the grains of Liaoza-48, Liaonuo-11, and Liaonian-3 were further identified. The results showed that all three sorghum grain extracts contained two major monomeric components: Apigeninidin and Luteolinidin. Among the three extracts, the extract of Liaoza-48 had the highest content of 3-DAs, Liaonuo-11 had the second highest content of 3-DAs, and Liaonian-3 had the lowest content of 3-DAs. Because of this, the 3-DAs extracted from Liaoza-48 demonstrated superior antioxidant activity to that of the 3-DAs extracted from Liaonuo-11 and Liaonian-3 as evidenced by higher scavenging activities for DPPH, ABTS^+^, and hydroxyl radicals accompanied by a lower IC_50_ value. These results provide technical guidance and a theoretical basis for the extraction and application of sorghum 3-DAs in the fields of food and medicine; this is beneficial for the high-value utilization of sorghum in China.

## Figures and Tables

**Table 1:** Response surface optimization design.

Level	Factors		
	*A:* Methanol/(*v*/*v*, %)	*B:* Solid-Liquid Ratio/(g/mL)	*C:* Extraction Time /min
−1	80	1:15	90
0	90	1:20	120
1	100	1:25	150

**Table 2:** Response surface analysis design and results.

No.	*A*	*B*	*C*	*Y:* 3-DAs Amount/(mg/g)
1	−1	−1	0	0.2082
2	0	0	0	0.2823
3	1	0	1	0.2712
4	0	1	−1	0.1576
5	−1	0	−1	0.1874
6	−1	0	1	0.1785
7	0	0	0	0.2728
8	0	0	0	0.2760
9	−1	1	0	0.1432
10	1	1	0	0.2648
11	0	−1	−1	0.1784
12	1	−1	0	0.2753
13	0	0	0	0.2813
14	0	−1	1	0.2427
15	0	1	1	0.1742
16	1	0	−1	0.2411
17	0	0	0	0.2677

**Table 3:** ANOVA for the response surface quadratic model (Cor Total: Corrected Total Sum of Squares).

Variance Source	Degree of Freedom	Sum of Squares	Mean Square	*F*-Value	*p*-Value
Model	9	0.0386	0.0043	35.78	<0.0001 **
A	1	0.0140	0.0140	116.99	<0.0001 **
B	1	0.0034	0.0034	28.29	0.0011 **
C	1	0.0013	0.0013	10.86	0.0132 *
AB	1	0.0007	0.0007	6.19	0.0417 *
AC	1	0.0004	0.0004	3.17	0.1183
BC	1	0.0006	0.0006	4.74	0.0659
A^2^	1	0.0005	0.0005	4.18	0.0803
B^2^	1	0.0076	0.0076	62.60	<0.0001 **
C^2^	1	0.0087	0.0087	72.84	<0.0001 **
Residual	7	0.0008	0.0001		
Lack of fit	3	0.0007	0.0002	6.29	0.0539
Pure error	4	0.0001	0.0000		
Cor total	16	0.0395			

*: *p* < 0.05, had a significant effect on the results; **: *p* < 0.01, had a very significant effect on the results.

## Data Availability

All of the data is contained within the article.

## Supplementary Materials

The following supporting information can be downloaded at: https://www.mdpi.com/article/10.3390/antiox12020468/s1, Table S1: Single-factor analysis; Table S2: Mobile phase gradient elution conditions; Table S3: Composition of DPPH reaction liquids; Table S4: Composition of ABTS^+^ reaction liquids; Table S5: Calculation of recovery rate of 3-DAs components in sorghum.
