# Supplementary material for: Extraction, Identification and Antioxidant Activity of 3-Deoxyanthocyanidins from Sorghum bicolor L. Moench Cultivated in China
Source: Antioxidants (Basel). 2023 Feb 12;12(2):468. doi: 10.3390/antiox12020468 (PMC9952376; doi:10.3390/antiox12020468)
Supplement: Supplementary file 1 [file antioxidants-12-00468-s001.zip › antioxidants-2193567-supplementary.pdf]

Supplementary Materials

Extraction, Identification and Antioxidant Activity of 3-deoxyanthocyanidins from *Sorghum bicolor* L. Moench Cultivated in China

Yanbei Wu, Yali Wang, Zhengyan Liu, and Jing Wang *

China-Canada Joint Lab of Food Nutrition and Health (Beijing), School of Food and Health, Beijing Technology and Business University (BTBU), 11 Fucheng Road, Beijing, 100048, China

* Correspondence:

Jing Wang, email: wangjing@th.btbu.edu.cn

1. Single-factor analysis

Table S1. Single-factor analysis.

Level	Factors			
	Methanol-Acetone /(v/v, %)	Solid-liquid ratio/(g/mL)	Extraction time/min	Extraction temperature/℃
1	60	1:5	30	25
2	70	1:10	60	30
3	80	1:15	90	35
4	90	1:20	120	40
5	100	1:25	150	45

2. Mobile phase gradient elution conditions

Table S2. Mobile phase gradient elution conditions.

Time/min	Mobile phase A/%	Mobile phase B/%	Flow rate/(mL/min)
0.01	90	10	1.0
5	90	10	1.0
10	70	30	1.0
20	40	60	1.0
25	80	20	1.0
30	90	10	1.0

A: 0.1% of formic acid aqueous solution; B: 0.1% of formic acid-acetonitrile solution.

3. Composition of DPPH reaction liquids

Table S3. Composition of DPPH reaction liquids.

Composition	Volume of reaction solution/μL		
	0	1	2
DPPH solution	600		600
Absolute alcohol	400	600	
Sample solution		400	400
Total volume	1000	1000	1000

4. Composition of ABTS$^+$ reaction liquids

Table S4. Composition of ABTS$^+$ reaction liquids.

Composition	Volume of reaction solution/μL	
	0	1
ABTS$^+$ solution	600	600
Ultrapure water	400	
Sample solution		400
Total volume	1000	1000

5. Calculation of recovery rate of 3-DAs components in sorghum

Table S5. Calculation of recovery rate of 3-DAs components in sorghum.

No.	Apigeninidin				Luteolinidin			
	Tested/ mg	Added/ mg	Recovery rate/%	RSD/ %	Tested/ mg	Added/ mg	Recovery rate%	RSD/ %
1	0.098	0.1	98.0	1.3%	0.098	0.1	98.0	1.5%
2	0.100	0.1	100		0.100	0.1	100	
3	0.195	0.2	97.5		0.198	0.2	99.0	
4	0.194	0.2	97.0		0.193	0.2	96.5	
5	0.484	0.5	96.8		0.493	0.5	98.6	
6	0.500	0.5	100		0.478	0.5	95.6	
